# bnglViz: online visualization of rule-based models

**DOI:** 10.1093/bioinformatics/btae351

**Published:** 2024-05-30

**Authors:** Noah Liguori-Bills, Michael L Blinov

**Affiliations:** Marine Earth and Atmospheric Sciences Department, North Carolina State University, Raleigh, NC 27695, United States; R. D. Berlin Center for Cell Analysis and Modeling, University of Connecticut School of Medicine, Farmington, CT 06030, United States

## Abstract

**Motivation:**

Rule-based modeling is a powerful method to describe and simulate interactions among multi-site molecules and multi-molecular species, accounting for the internal connectivity of molecules in chemical species. This modeling technique is implemented in BioNetGen software that is used by various tools and software frameworks, such as BioNetGen stand-alone software, NFSim simulation engine, Virtual Cell simulation and modeling framework, SmolDyn and PySB software tools. These tools exchange models using BioNetGen scripting language (BNGL). Until now, there was no online visualization of such rule-based models. Modelers and researchers reading the manuscripts describing rule-based models had to learn BNGL scripting or master one of these tools to understand the models.

**Results:**

Here, we introduce bnglViz, an online platform for visualizing BNGL files as graphical cartoons, empowering researchers to grasp the nuances of rule-based models swiftly and efficiently, and making the exploration of complex biological systems more accessible than ever before. The produced visualizations can be used as supplemental figures in publications or as a way to annotate BNGL models on web repositories.

**Availability and implementation:**

Available at https://bnglviz.github.io/.

## 1 Introduction

The rule-based modeling approach (Hlavacek *et al.* 2006) provides a compact description of molecules, molecular interactions, and their effects in the form of templates for possible chemical species and reactions. These templates are based on the description of biomolecules as containers that have multiple sites (e.g. molecular binding sites such as SH2 domains, post-translational modification sites, tyrosine residues or ITAMs), their states (such as phosphorylated on unphosphorylated states of a tyrosine), and connectivity of molecules through explicit binding among molecular binding sites. Thus, the rule-based modeling approach enables the representation of intricate details in biochemical processes and provides high-level expressiveness in describing molecular interactions and reactions. The actual chemical species and reactions are generated either deterministically, using BioNetGen (Blinov *et al.* 2004, Harris *et al.* 2016) simulation engine, or stochastically, using agent-based modeling with NFSim (Sneddon *et al.* 2011).

Rule-based modeling using BioNetGen was adopted by a variety of software tools, such as Virtual Cell (Loew and Schaff 2001, Moraru *et al.* 2008), PySB (Lopez *et al.* 2013), Smoldyn (Andrews 2017), and MolClustPy (Chattaraj *et al.* 2023). These tools use BioNetGen simulation engine to simulate rule-based models described in BioNetGen Language (BNGL, Faeder *et al.* 2009).

Rule-based modeling has been used in studies of multiple signaling pathways, insights into structure-driven signaling dynamics, analysis and molecular cluster formations, and more. Every year, numerous rule-based models are published, and the accompanying BNGL code for these models is typically included in supplementary materials (just for 2023 these are Jaruszewicz-Błońska *et al.* 2023, Korwek *et al.* 2023, Zhang *et al.* 2023, MacKenzie *et al.* 2024). The partial list of models with their visualizations is available at the website http://bnglviz.github.io/, starting with the first rule-based models by Goldstein *et al.* (2002) and Faeder *et al.* (2003) and including the recent publications in high-profile journals, such as by McMillan *et al.* (2021), Nosbisch *et al.* (2022), and Korwek *et al.* (2023).

However, BNGL is not fully human-readable, and models encoded in BNGL are difficult to comprehend without a steep learning curve. Reading BNGL, understanding the molecular connectivity, and visualizing the model requires extensive experience in scripting or running special client-based software tools such as Virtual Cell software that enable the design of rule-based models without scripting.

Here, we present a web tool for online graphical visualization of rule-based models. It significantly eases the burden of understanding the scripting BioNetGen language. Any BNGL model can be loaded and fully visualized in a colorful cartoon-style way, following graphical notations introduced within a popular modeling and simulation framework Virtual Cell (Schaff *et al.* 2016, Blinov *et al.* 2017) that are based on cartoons first introduced in Faeder *et al.* (2005) and Blinov *et al.* (2006). The generated visualizations can be used as supplemental figures in publications or as a way to annotate BNGL models on web repositories.

## 2 Visualization of BNGL code with bnglViz

**Figure 1.**
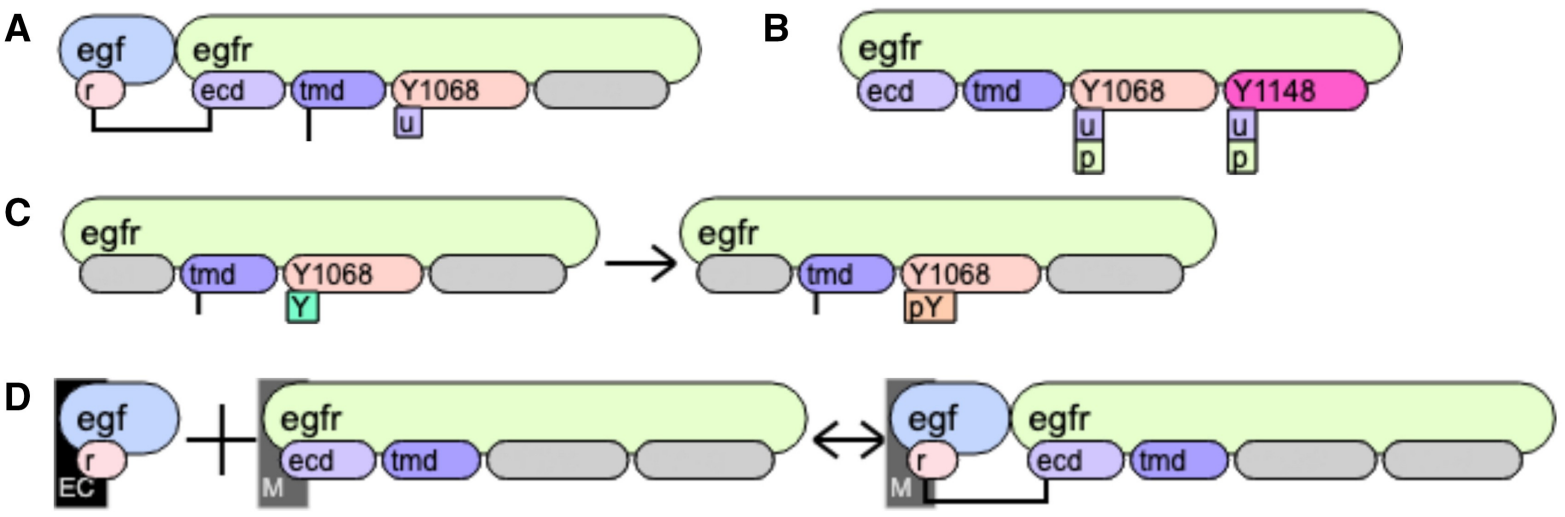
Visualization of rule-based models using bnglViz. (A) Description of a molecular template. Two molecules “egf” and “egfr” are connected at the two sites “r” and “ecd,” respectively. The transmembrane domain (“tmd” site) of “egfr” must be bound to some other molecule, indicated by a vertical line. “Y1068” site of “egfr” is unphosphorylated (denoted by a square with a letter “u”) and unbound, while the state of “Y1148” is not defined and this site may be bound or unbound. (B) The full description of an “egfr” molecule. It has four sites: extracellular “ecd,” transmembrane “tmd” and two tyrosine “Y1068” and “Y1148.” Each of the two tyrosine can be in two possible states: unphosphorylated (“u”) and phosphorylated (“p”). (C) The simple rule of phosphorylation of “Y1069” residue of “egfr” receptor by another receptor bound to it. The trans-membrane domain of egfr (“tmd” site) must be bound for the state of “Y1068” to change from unphosphorylated (“u”) to phosphorylated (“p”). (D) Adding locations to rule-based description. “egf” ligand is located in a volumetric extracellular compartment (“EC”), while “egfr” receptor is located on a membrane “M.” The resulting complex is located on a membrane “M.” For the reaction to proceed, the trans-membrane domain (“tmd”) must be unbound.

A model encoded in BNGL (such as taken from supplemental material) can be loaded at the website, and then all molecules, species, reaction rules, and observables will be displayed on the same page. We use the conventions introduced in VCell (Schaff *et al.* 2016). BNGL is a scripting language that operates with expressions like

egf(r!1).egfr(ecd!1,tmd!+,Y1068∼u,Y11148!?)

to describe molecular configurations. The expression above depicts two molecules “egf” and “egfr,” connected via a bond between the site “r” of “egf” and the site “ecd” of “egfr.” The molecule “egfr” has three more sites: “tmd” that must be bound to another site, “Y1068” that is phosphorylated and unbound from any other molecule, and “Y1148” that may be in any phospho-state and may be either bound or unbound. bnglViz visualizes this expression in Fig. 1A. Figure 1B shows the possible states of sites in “egfr” molecule. Contrary to BNGL script where all elements not important for the rule are omitted, bnglViz shows all sites of molecules, but the sites not participating in the reaction rule are shown as grey shapes. For example, the reaction rule that phosphorylates the site “Y1068” while “egfr” receptor is bound to another molecule at its transmembrane site “tmd” is shown in BNGL as

egfr(tmd!+,Y1068∼Y) → egfr(tmd!+,Y1068∼pY) 

(meaning the states of “r” and “Y1148” sites are irrelevant to the outcome of the reaction rule), while its visualization is demonstrated in Fig. 1C.

Note that all nonvisual parts of BNGL code such as parameter's block and actions are omitted in visualization for clarity, but all comments for molecules, species, rules and observables are displayed.

## 3 Limitations

First, bnglViz should not be used for code or model validation. While looking at visualization may help catch typos, the tool does not match names within different parts of the code. No code validation is provided: the BNGL is visualized as is written. Only some errors in the code are noted and provided to the user, such as when an observable has no defined type.

Second, BioNetGen works with compartments in two different ways: cBNGL (compartmental BNGL, Harris *et al.* 2009) extension assigns compartments to individual sites, while rule-based models generated with VCell have a compartment assigned to each reaction and product pattern in reaction rules (Blinov *et al.* 2017). bnglViz does not support cBNGL, while visualizing compartments in BNGL generated with VCell (Fig. 1D).

Next, BNGL is still under development and different teams may introduce different features (such as compartmental description in VCell versus cBNGL). Please contact authors for support of new features.

Finally, bnglViz does not provide a global visualization of the model that shows how the rules or other model elements interact (Tiger *et al.* 2012, Cheng *et al.* 2014, Forbes *et al.* 2017, Sekar *et al.* 2017).

## 4 Implementation

bnglViz is implemented as native JavaScript on a static website hosted using GitHub pages. All of the source code is available at https://github.com/bnglViz/bnglViz.github.io. The color of molecules and sites is hashed from their name such that two molecules that share a name will always be a consistent color. A dark mode button is provided for screenshot convenience.

## 5 Conclusions

We have demonstrated the use of bnglViz to visualize published rule-based models encoded in BNGL. More than 30 published models implemented in BioNetGen are available on our website at https://bnglviz.github.io/examples.html as both BNGL code and bnglViz-generated visualization. Several other rule-based languages have similar features as BNGL. These include Kappa Language (Boutillier *et al.* 2018), Simmune (Zhang *et al.* 2013), ML-rules (Maus *et al.* 2011), and rule-based SBML extension (Zhang and Meier-Schellersheim 2018). bnglViz could, in the future, be adapted to visualize their model specification languages as well.
